# The impact of age on the survival outcomes of hepatocellular carcinoma patients after transarterial chemoembolization: A systematic review and meta-analysis

**DOI:** 10.12669/pjms.41.4.11718

**Published:** 2025-04

**Authors:** Xue Du, Xiaoting Zhang

**Affiliations:** 1 Xue Du Department of Hepatobiliary Pain Interventional Diagnosis and Treatment Center, Lishui Central Hospital, Lishui, Zhejiang Province 323000, P.R. China; 2 Xiaoting Zhang Department of Hematology, Lishui Central Hospital, Lishui, Zhejiang Province 323000, P.R. China

**Keywords:** Hepatocellular carcinoma, Meta-Analysis, Older adults, Transarterial chemoembolization

## Abstract

**Background & Objective:**

The efficacy of transarterial chemoembolization (TACE) in different populations of hepatocellular carcinoma (HCC) patients is still unclear. This meta-analysis explores the impact of TACE on survival outcomes in elderly versus younger patients with HCC, considering regional variations and heterogeneity among studies.

**Methods:**

Nineteen studies involving 30,093 participants were systematically reviewed from January 1964 to January 2024. Data were pooled using random-effects models to calculate hazard ratios (HRs) and odds ratios (ORs) with 95% confidence intervals (CI) for overall survival and survival rates, respectively. Subgroup analyses were conducted based on age cut-offs and geographical regions to assess the effect of these variables on treatment outcomes.

**Results:**

Pooled HR for overall survival did not show a significant difference between elderly and younger patients (HR = 1.00; 95% CI: 0.98 to 1.02), with similar findings for survival rates (OR = 0.82; 95% CI: 0.46 to 1.45). Substantial heterogeneity was observed (I² = 78.0% for HR and 94.3% for OR), with notable regional differences indicating lower survival odds in European studies compared to Asian ones. No significant effect (OR = 0.95) was detected in prospective studies, while retrospective studies indicated a significant reduction in survival rates in elderly patients (OR = 0.35).

**Conclusion:**

TACE appears to be equally effective in elderly and younger HCC patients. However, significant regional differences and study heterogeneity suggest the need for personalized treatment approaches. Further research is required to understand the underlying causes of these variations and to optimize TACE protocols.

## INTRODUCTION

Hepatocellular carcinoma (HCC) is a major global health challenge, and ranks as the sixth most common cancer and the third leading cause of cancer-related death worldwide.^1^ The incidence of HCC is intrinsically linked to chronic liver diseases, often arising in the context of cirrhosis due to viral hepatitis, alcohol consumption, or non-alcoholic steatohepatitis.^2^ȓ^4^ HCC management is complex and multifaceted, involving a range of therapeutic modalities tailored to the stage of the disease, underlying liver function, and patient factors such as comorbidities and performance status.^5^ Transarterial chemoembolization (TACE) has emerged as a cornerstone in the treatment of intermediate-stage HCC.^6^

TACE is based on the targeted delivery of chemotherapeutic agents directly to the tumour bed in combination with embolizing agents that restrict tumour’s blood supply.^7^ This technique has been shown to prolong survival in patients with unresectable HCC, making it a widely accepted practice in the treatment of HCC in patients with intermediate-stage disease and relatively preserved liver function. However, gradual aging of the global population presents new challenges in the management of HCC.^8^ The prevalence of HCC increases with age, and majority of HCC patients are diagnosed at the advanced age^9^ that is associated with a distinct set of challenges, including increased comorbidities, diminished physiological reserves, and altered pharmacokinetics, all of which can influence treatment outcomes.^10^,^11^ Furthermore, older age may be associated with a different tumour biology, potentially affecting the response to therapies such as TACE.

However, the impact of age on the outcomes of TACE remains incompletely understood. Studies have reported varying results, with some reports suggesting comparable efficacy and safety of TACE in older and younger patients, while others indicating increased complications or reduced survival in elderly population.^12^ȓ^14^ These conflicting results highlight the need for a systematic evaluation of existing evidence to understand the true impact of age on TACE outcomes., especially in the context of comprehensive nursing care. Nurses play a pivotal role in monitoring treatment responses, managing side effects, and providing tailored support that addresses the unique needs of older patients. Therefore, understanding differences in outcomes of TACE in HCC patients of different age groups are essential in order to develop and implement nursing care plans that optimize patient comfort, adherence to treatment, and overall quality of life. This review aimed to critically evaluate and synthesize the existing literature on the impact of older age on the outcomes in patients with hepatocellular carcinoma undergoing TACE.

## METHODS

The review has included studies involving adult patients (aged 18 years and older) diagnosed with hepatocellular carcinoma. The primary exposure of interest is older patients undergoing TACE as a treatment for HCC. The comparator group consists of the patients within the same studies who are younger and receiving TACE for HCC. Studies without a clear age demarcation or comparison were excluded. The primary outcomes of interest were overall survival rates, duration of survival and treatment-related adverse events or complications (Grade-III or above). The review has included randomized controlled trials, cohort studies, case-control studies, and observational studies. Case reports, editorials, reviews, and animal studies were excluded.

### Information sources and search strategy:

The search for relevant studies were conducted in the following electronic databases, registers, and other sources: MEDLINE (via PubMed), EMBASE, Cochrane Central Register of Controlled Trials, Web of Science, Scopus, ClinicalTrials.gov. Manual search of reference lists from relevant studies and review articles was done to identify additional studies not captured by electronic searches. Targeted search of websites of relevant professional organizations and societies in the field of oncology and hepatology, such as American Society of Clinical Oncology (ASCO) and the European Association for Study of Liver (EASL) was done. All databases and other sources were searched from their inception (January 1964) to January 2024 ensuring comprehensive and up-to-date review of the available literature without any language filters. The database search was executed from September to October 2023 and then updated again at the end of January 2024.

### Study Selection Process:

Two independent reviewers meticulously examined titles, abstracts and key terms of each study. Subsequently, full-text articles were assessed using pre-established inclusion criteria. In cases of disagreement, a consensus was reached through collaborative discussion. The entire review process was rigorously documented, aligning with the standards set by the PRISMA guidelines.^15^

### Data Extraction Methodology:

Key information was extracted from the selected studies by the lead researcher, and included publication details (such as extraction date, study titles, and author names), and methodological elements (study design, participant demographics, and contextual details). Specific focus was given to extracting data such as the number of subjects in each study group, baseline and final outcome measures, inclusion and exclusion criteria, details of interventions, comparison groups, and the length of follow-up periods.

### Risk of Bias Assessment:

Risk of bias was assessed using the Newcastle-Ottawa Scale (NOS).^16^ The NOS assesses the quality of observational studies based on three broad perspectives: selection of study groups, comparability of groups, and ascertainment of either the exposure (for case-control studies) or the outcome of interest (for cohort studies). Each study can be awarded a maximum of nine stars, representing high methodological quality.

### Data synthesis and analysis:

For the meta-analysis, STATA version 14.2 (StataCorp, College Station, TX, USA) was used. Natural logarithm of the hazard ratio (lnHR) along with its standard error (SE) were used to analyse time-to-event data, such as overall survival (OS). Initially, hazard ratios (HRs) and their 95% Confidence Intervals (CIs) were extracted from each study. Subsequently, the ln(HR) for each study’s HR estimate was calculated. Pooled effect size were then computed using ln(HR) values and their corresponding SEs. A random-effects model was applied to account for variability. Results were reported as pooled HRs with 95% CIs, and graphically represented through forest plots.^17^

For binary outcomes, numbers of participants experiencing these outcomes in both exposed and non-exposed groups were recorded. From these data, we derived pooled odds ratios (ORs) with 95% CIs. For continuous outcomes, mean and SD in each group was obtained and then pooled standardized mean difference (SMD) with 95%CI was reported.^17^ Heterogeneity among the studies was assessed using I^2^ statistic and chi-square test for heterogeneity. Subgroup analyses were done based on age cut-offs of patients, study region and study design. Publication bias assessment was done using Egger’s test and funnel plot.

## RESULTS

**Fig.1:**
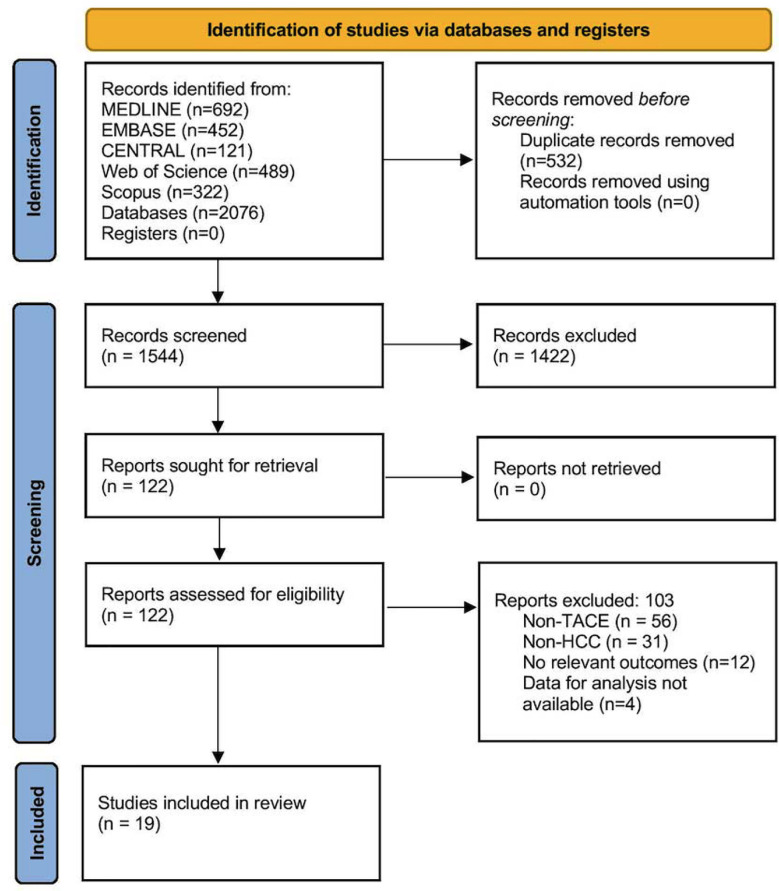
PRISMA flowchart.

Overall, 1932 records were retrieved by search across the databases. After deduplication, 1544 records underwent primary title, abstract and key terms screening, and 122 full texts were retrieved to check final eligibility. Finally, 19 studies were included (Fig.1).^12^–^14^,^18^–^33^

### Characteristics of the included studies:

As shown in Table-I, the review included 19 studies, conducted across diverse range of countries. Most studies were conducted in China, followed by European countries like Italy, Israel, Germany, Spain, and Switzerland. These studies collectively analysed 30,093 participants, primarily focusing on patients with HCC undergoing TACE. Studies varied significantly in sample sizes, ranging from 38 to 15,186 participants, with a majority reporting comorbid conditions such as cirrhosis, and hepatitis B and C infections. The male to female ratio across studies was notably skewed towards male patients. In terms of study quality, five studies were rated high, six studies were moderate, and seven studies were low (Table-I).

**Table-I:** Characteristics of the included studies (N=19).

	Study location, design and quality	Study Participants description	Comorbidity details	Child Pugh A / B	Male: Female ratio	Mean age in years
Biselli M et al. 1997	Italy Cohort study High	103 patients with HCC aged 65 years and over underwent EAT. And 65 patients aged 64 or under with HCC who underwent EAT.	Cirrhosis: I – 35, C- 62 HBV: I – 5, C – 20 HCV: I – 31, C - 40	A – 42* B – 44 C - 11	84:19	NR
Cohen M J et al. 2013	Israel Prospective cohort study Low	102 patients diagnosed with HCC between 2000 and 2010 who underwent TACE were included.	Cirrhosis: Eld – 22, Int -41, Youn- 35 HBV: Eld – 2, Int -7, Youn -12 HCV: Eld – 18, Int -29, Youn -21	A – 85 B – 14 C - 3	27:75	NR
Cohen M J et al. 2014	Spain, Italy, China and Israel Cohort design analysis and High	Medical literature reporting prognosis following TACE among 548 patients with HCC, which included data stratification according to age groups. Patients diagnosed and treated between 1988 and 2010 were included in the analysis.	Cirrhosis: 325 HBV: 184 HCV: 227	A – 378* B – 145 C - 23	417:131	NR
Frundt T W et al. 2022	Germany, Single centre cohort study, moderate	656 Patients with a confirmed diagnosis of HCC with liver cirrhosis or had undergone orthotopic liver transplantation who were treated at the University Medical Centre Hamburg-Eppendorf between 2008 and 2017 were included in this study.	HBV: Eld – 17, Int -32, Youn -52 HCV: Eld – 40, Int -51, Youn -79	A – 321* B – 147 C - 65	542:114	Median: YP –56(23-60) IP – 66(61-70) EP -75(71-87)
Golfieri R et al. 2013	8 European centers, Cohort study, Moderate	325 Elderly and younger patients with unresectable HCC who received radioembolization between 25 September, 2003 and 17 December, 2009.	Cirrhosis: I – 104, C- 151 HBV: I – 10, C – 32 HCV: I – 59, C - 85	A – 268 B – 57	265:60	NR
Heng-jun G et al. 2013	China, Cohort study, Low	1516 Patients who underwent TACE as an initial treatment for HCC from October 2000 to October 2009 at the Sun Yat-Sen University Cancer Center	HBV: 502	A – 723* B – 111	1357:159	Median – 47(23 - 84)
Hu H et al. 2015	China, Cohort study, High	88 advanced HCC patients, with vascular invasion and/or distant metastasis corresponding to Barcelona Clinic Liver Cancer (BCLC) stage C, from March 2009 to November 2013	Cirrhosis: I – 14, C- 42 HBV: I – 13, C – 41 HCV: I – 3, C - 5	A – 61 B – 27	75:13	Mean age Elderly - 76 yrs; range, 70–83 yrs Non elderly - 57 yrs; range, 31–69 yrs
Kong J et al. 2018	China, Retrospective case control study and High	522 Patients who underwent TACE or conservative management as initial treatment in Fourth Hospital of Hebei Medical University from January 2002 to December 2010, was investigated.	Cirrhosis: I -311, C -159	A – 309 B – 152 C - 61	439:83	Age ranges from 18 to 94
Lee H A et al. 2023	Seoul, Korea, Cohort study, Moderate	15186 Patients above 18 years of age with HCC registered in the KPLCR between January 2008 and December 2017 were included.	HBV: I – 1971, C – 7094 HCV: I – 1031, C - 535	A – 10505* B – 3267 C - 789	12034:3152	Median age Elderly - 72 (68-77) yrs Non elderly - 54 (49-59) yrs
Liu P et al. 2014	Taiwan, Cohort study, Moderate	3082 newly diagnosed HCC patients in Taipei Veterans General Hospital from 2002 to 2013 were involved in the study.	HCV: I – 278, C - 662	A – 2250 B – 695 C - 137	2371:711	Mean age Elderly – 80.3 ±4.1 yrs, Younger – 58.6 ±10.5 yrs
Mirici-Cappa F et al. 2009	Italy, Retrospective cohort study and nested case control study, Moderate	1718 Patients with HCC in ten medical institution was included in the study at the time duration of January 1987 to December 2004	Cirrhosis: I – 578, C- 1040 HBV: I – 45, C – 130 HCV: I – 381, C - 518	A – 1044* B – 506 C - 141	1256:462	Mean age Elderly – 74.6 ±3.9 yrs, Younger – 60.8 ±6.7 yrs
Mosconi C et al. 2020	Italy, Cohort study, Low	225 Patients with HCC who underwent lipiodol based TACE as the first line treatment between January 2011 and December 2016 were included	HBV: I – 5, C – 19 HCV: I – 53, C - 72	A – 155 B – 70	171:54	Mean age Elderly – 60 ±7 yrs, Younger – 75 ±4 yrs
Nishikawa H et al. 2014	Japan	150 Patients with HCC undergoing TACE at the duration between December 2003 to December 2012	Not reported	A – 105 B – 45	97:53	Mean age Elderly – 80.8 ±4.2 yrs, Younger – 65.7 ±5.6 yrs
Poon R T P et al. 1999	China, Cohort study, Moderate	1338 Newly diagnosed HCC patient who have never undergone any treatment were included in the study at the duration of January 1989 to December 1997	Not reported	A – 958 B – 296 C - 84	1148:190	Mean age Elderly – 75 ±4 yrs, Younger – 53 ±11 yrs
Roth G S et al. 2022	Switzerland, Cohort study, Low	271 Patients at age group > 18 who received a first TACE for HCC at the Grenoble-Alpes University Hospital from 1 January 2012 to 2 March 2017	Cirrhosis: I – 63, C- 168	A – 176* B – 49 C - 4	249:22	Median age Elderly - 75 (71-79) yrs Non elderly - 62 (56-65) yrs
Xiao J et al. 2014	China, Cohort study, Moderate	2493 Patient with HCC and with initial treatment on TACE no extra hepatic metastasis were included in the study from the duration of January 2004 to December 2008.	HBV: I – 349, C – 1033 HCV: I – 6, C - 19	A – 519 B – 1974	2134:359	Mean age Elderly – 67 ±5.4 yrs, Younger – 45.6 ±8.9 yrs
Yang Q et al. 2018	China, Cohort study, High	91 HCC patients treated by DEB-TACE therapy	Not reported	Not reported	Not reported	Not reported
Yau T et al. 2009	China, Cohort study, Moderate	1040 Advanced hepatocellular carcinoma (HCC) patients who received TACE at the authors’ center were analyzed Between 1989 and 2006	HBV: I – 112, C – 703 HCV: I – 21, C - 43	A – 839 B – 191 C - 10	858:182	Median age Elderly - 75 (71-78) yrs Non elderly - 58 (17-70) yrs
Zhang L et al. 2023	China, Cohort study, Moderate	198 HCC patients who underwent TACE as their initial treatment at our institution between January 2015 and December 2021 were involved	HBV: I – 29, C – 128 HCV: I – 7, C - 6	A – 173 B – 25	159:39	Mean age Elderly – 81.6 ±1.8 yrs, Younger – 60.8 ±10 yrs

C – Control; I – Intervention; NR – Not reported; NA – Not applicable; RCT – randomized controlled trial; USA – United States of America;

* - Missing data.

### Overall survival (reported as HR):Overall

**Fig.2:**
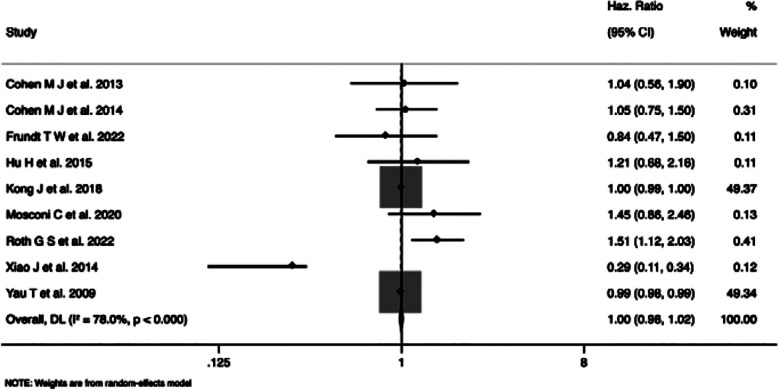
Forest plot showing the difference in overall survival (in terms of Hazard ratio) between young and elderly hepatocellular carcinoma patients undergoing transarterial chemoembolization.

Meta-analysis of nine studies that reported overall survival did not find a statistically significant overall effect of the TACE on the survival of patients, with a pooled HR of 1.00 (95%CI: 0.98-1.02) and a nonsignificant overall effect (z=-0.406, p=0.685) (Fig.2). However, substantial heterogeneity was observed (I² = 78.0%, Cochran’s Q=36.36, p<0.001). The meta-analysis differentiated studies by age cut-offs, revealing no significant difference in HRs between younger (60/65 years cut-off, pooled HR=0.56, 95%CI: 0.17 to 1.87) and older age groups (70, 75, or 80 years cut-off, pooled HR=1.12, 95%CI: 0.94 to 1.32). The meta-analysis stratified by region indicated no significant differences in HRs between studies conducted in Europe and Asia.

### Overall survival rates (reported as dichotomous outcome):

**Fig.3:**
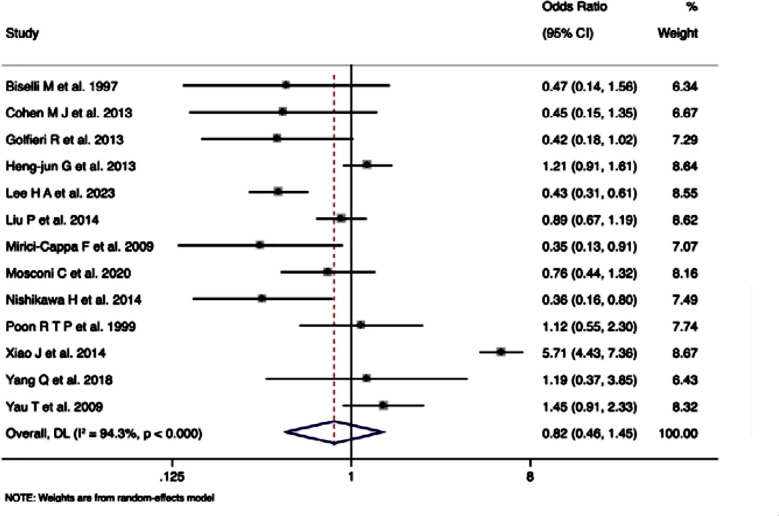
Forest plot showing the difference in overall survival rates (in terms of dichotomous outcomes) between young and elderly hepatocellular carcinoma patients undergoing transarterial chemoembolization.

Thirteen studies with 8,408 participants reporting overall survival rates showed pooled OR of 0.82 (95%CI: 0.46-1.45), indicating no significant difference between overall survival rates in elderly and younger HCC patients undergoing TACE (z=-0.687, p=0.492) (Fig.3) with substantial heterogeneity (I²=94.3%, Cochran’s Q=210.82, p<0.0001).

Analysis, stratified by age groups, showed no significant difference in ORs for survival outcomes between the 60/65 years cut-off subgroup (OR=1.14, 95%CI: 0.36-3.62) and the 70, 75, or 80 years cut-off subgroup (OR=0.72, 95%CI: 0.51-1.03). Meta-analysis stratified by region showed a significant reduction in ORs in studies conducted in Europe (OR=0.55, 95% CI: 0.38-0.80, p=0.002), while no such effect was reported for Asian studies (OR=1.10, 95%CI: 0.53-2.27, p=0.804).

Meta-analysis that differentiated studies based on their design revealing no significant effect in prospective studies (OR=0.95, 95%CI: 0.51-1.74, p=0.858). However, there was significant reduction in survival rates in elderly compared to younger patients in retrospective studies (OR=0.35, 95%CI: 0.19-0.66, p=0.001). Funnel plot was slightly asymmetrical, but not statistically significant (p=0.11).Top of Form

### Duration of survival:

**Fig.4:**
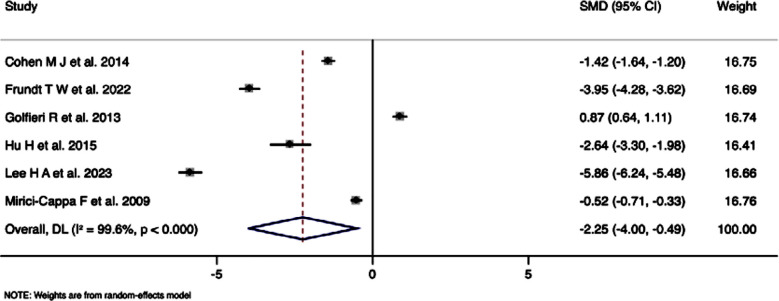
Forest plot showing the difference in duration of survival between young and elderly hepatocellular carcinoma patients undergoing transarterial chemoembolization

Six studies with 2,489 participants, assessing the duration of survival in elderly and young patients, pooled SMD was -2.25 (95%CI: -4.00 to -0.49), indicating statistically significant difference (p=0.012) (Fig.4). However, analysis revealed extremely high heterogeneity (I²=99.6%), with Cochran’s Q statistic of 1223.97 (p<0.001).

### Treatment related adverse events or complications (Grade-III and above):Top of Form

**Fig.5:**
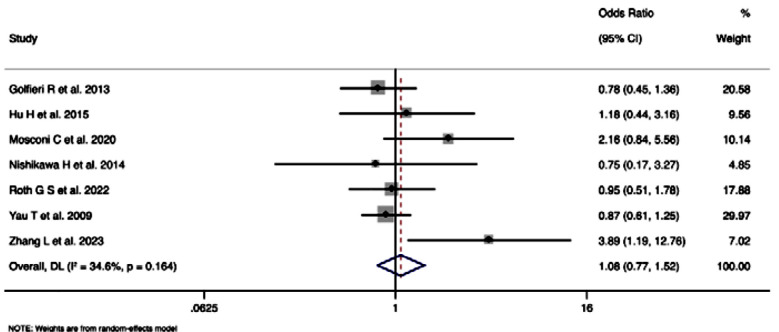
Forest plot showing the difference in treatment related adverse events or complications (Grade-III or above) between young and elderly hepatocellular carcinoma patients undergoing transarterial chemoembolization.

Seven studies with 2,281 participants, assessing treatment-related adverse events/complications of Grade-III or above, pooled OR was 1.08 (95%CI: 0.76-1.52), indicating no statistically significant difference in the odds of experiencing such events between two groups (z=0.429, p=0.668) (Fig.5). Heterogeneity among included studies was moderate (I²=34.6%), with Cochran’s Q statistic of 9.18 (p=0.164).

## DISCUSSION

This meta-analysis synthesized data from 19 studies involving 30,093 participants to assess the difference in impact of TACE on elderly and young patients with HCC. The findings reveal no significant overall effect of TACE on survival rates when comparing elderly to younger patients, with pooled HR of 1.00 and OR of 0.82 for overall survival and survival rates, respectively.

Our results align with some previous studies that found no significant difference in survival outcomes between elderly and younger HCC patients undergoing TACE.^20^–^25^,^30^,^32^ However, our study contrasts with other research suggesting that age significantly influences TACE outcomes, highlighting the ongoing debate within the field. Notably, the significant reduction in survival odds in European studies compared to Asian studies suggests regional variations in TACE efficacy or patient selection, which warrants further investigation.

The observed regional differences in TACE outcomes could be reflective of differences in medical practices, access to healthcare, and the technological advancement of medical facilities. European healthcare systems, with their generally uniform healthcare policies and high levels of access to advanced medical treatments, might offer a different context for TACE outcomes compared to Asian countries, where healthcare systems vary widely from country to country.^34^ These disparities could affect not only the implementation of TACE but also post-treatment care and monitoring, potentially influencing survival rates. Understanding these differences is crucial for developing global guidelines that can be adapted to regional contexts, ensuring that all patients have access to the best possible care.

The observed lack of significant effect across age groups might be due to the advanced therapeutic techniques and supportive care that minimize the impact of age on TACE outcomes. Moreover, the observed high heterogeneity and regional differences suggest that factors such as healthcare infrastructure, patient management protocols, and genetic or lifestyle differences might influence the efficacy of TACE across populations. The study’s strengths include its large sample size and the comprehensive nature of the analysis, incorporating a diverse range of studies from various regions. The use of both HRs and ORs to assess survival outcomes provides a thorough understanding of the impact of TACE on HCC patients.

The substantial heterogeneity found in this analysis underscores the complexity of treating HCC with TACE. Factors contributing to this heterogeneity could include differences in tumour staging at the time of treatment, liver function status, and the presence of underlying conditions such as cirrhosis or viral hepatitis, which are prevalent among HCC patients. Additionally, variations in TACE technique, such as the use of drug-eluting beads versus conventional TACE, and differences in chemotherapeutic agents used, could significantly impact outcomes.^35^ Future meta-analyses should strive to categorize studies based on these variables to provide more nuanced insights into the factors that most significantly affect patient outcomes following TACE.

The lack of significant difference in survival outcomes between elderly and younger patients undergoing TACE has important implications for clinical practice. It suggests that age alone should not be a determining factor in the decision to proceed with TACE for HCC patients. Nursing care plays a crucial role in the management of patients with HCC undergoing TACE, particularly considering our findings. Nurses, as key members of the multidisciplinary care team, must adapt their care strategies to reflect the nuanced understanding that age alone does not dictate TACE outcomes. This involves personalized patient education, vigilant monitoring for treatment-related complications, and tailored support to address the individual needs of each patient, thereby optimizing the therapeutic benefits of TACE across diverse age groups. Instead, a comprehensive assessment of an individual’s overall health status, liver function, and tumour characteristics should guide treatment decisions. This approach aligns with the trend towards personalized medicine, where treatments are tailored to the specific needs of each patient rather than based on broad demographic categories.^36^

The findings suggest that TACE remains a viable treatment option for HCC patients regardless of age, emphasizing the importance of individualized patient care over age-based treatment selection. The significant regional differences highlight the need for standardized protocols and further research to optimize TACE outcomes globally. Moreover, the variability in outcomes underscores the importance of considering patient-specific factors in treatment planning.

Future research should aim to explore factors contributing to the observed heterogeneity and regional differences in TACE outcomes. Specifically, studies investigating the role of healthcare infrastructure, patient management practices, and the impact of different TACE techniques on survival outcomes across various populations are needed. Moreover, research focusing on the optimization of TACE protocols to improve survival outcomes and minimize adverse events is crucial. Finally, studies assessing the cost-effectiveness of TACE in different regions could provide valuable insights for healthcare policy and resource allocation.

### Limitations

Substantial heterogeneity among the included studies may complicate the interpretation of the pooled results. The lack of data on specific TACE techniques and the absence of a publication bias assessment due to the limited number of studies in certain analyses also restrict the conclusiveness of the findings.

## CONCLUSION

This meta-analysis demonstrates that TACE does not significantly affect survival outcomes when comparing elderly to younger HCC patients, with notable heterogeneity and regional variations in study results. While age may not be a critical factor in determining the suitability of TACE for HCC patients, individualized treatment planning that considers a wide range of patient and regional characteristics is essential.

### PROSPERO registration number: CRD42024504968.

### Authors’ contributions:

**XD:** Study design, literature search and manuscript writing.

**XD and XZ:** Data collection, data analysis, interpretation and critical review.

**XD:** Manuscript revision and validation, critical analysis.

All authors have read, approved the final manuscript and are and is responsible for the integrity of the study.
